# Comparison of Visual and automated assessment of Ki-67 proliferative activity and their impact on outcome in primary operable invasive ductal breast cancer

**DOI:** 10.1038/bjc.2011.569

**Published:** 2012-01-03

**Authors:** Z M A Mohammed, D C McMillan, B Elsberger, J J Going, C Orange, E Mallon, J C Doughty, J Edwards

**Affiliations:** 1 Academic Unit of Surgery, College of Medical, Veterinary and Life of Sciences- University of Glasgow, Royal Infirmary, Glasgow G31 2ER, UK; 2 University Department of Pathology, College of Medical, Veterinary and Life of Sciences- University of Glasgow, Royal and Western Infirmaries, Glasgow, UK; 3 Unit of Experimental Therapeutics, Institute of Cancer, College of Medical, Veterinary and Life Sciences, University of Glasgow, Royal and Western Infirmary, Glasgow, G11 6NT, UK; 4 Department of Surgery, Western Infirmary, Glasgow, G11 6NT, UK

**Keywords:** primary invasive breast cancer, visual counting method, automated counting method, survival

## Abstract

**Background:**

Immunohistochemistry of Ki-67 protein is widely used to assess tumour proliferation, and is an established prognostic factor in breast cancer. There is interest in automating the assessment of Ki-67 labelling index (LI) with possible benefits in handling increased workload, with improved accuracy and precision.

**Patients and methods:**

Visual and automated assessment of Ki-67 LI and survival were examined in patients with primary operable invasive ductal breast cancer. Tissue microarrays (*n*=379 patients) immunostained for Ki-67 were scored visually and automatically with the Slidepath Tissue IA system.

**Results:**

Visual and automated Ki-67 LI were in excellent agreement (ICCC=0.96, *P*<0.001). On univariate analysis, visual (*P*<0.001) and automated Ki67 LI (*P*<0.05) were associated with cancer-specific survival in patients with invasive ductal breast cancer overall and in patients who received endocrine therapy (Tamoxifen) (*P*<0.01 for visual and *P*<0.05 for automated scoring).

**Conclusion:**

Automated assessment of Ki-67 LI would appear to be comparable to visual Ki-67 LI. However, automated Ki-67 LI assessment was inferior in predicting cancer survival in patients with breast cancer, including patients who received Tamoxifen.

Breast cancer accounts for 22% of all female cancers (Parkin *et al*, 2001). More than 42 000 women in the UK are diagnosed with breast cancer each year and approximately 80% survive at least 5 years (Cancerstats, 2008).

Tumour progression is influenced by tumour cell proliferation, which can be estimated by measuring the expression of the nuclear antigen Ki-67. Ki-67 expression is tightly linked to the cell cycle (Scott *et al*, 1991; McCormick *et al*, 1993), but does not appear to be expressed during DNA repair, and Ki-67 has been used to identify good and poor prognostic categories in invasive breast cancer (Fitzgibbons *et al*, 2000). Several recent studies have reported an association between higher Ki-67 proliferative activity and poorer recurrence-free (Goldhirsch *et al*, 2007; Viale *et al*, 2008; Jung *et al*, 2009) and cancer-specific survival (de Azambuja *et al*, 2007; Al Murri *et al*, 2008; Yerushalmi *et al*, 2010). The Ki-67 proliferative activity has also been reported to be associated with the clinical response to chemotherapy (Goldhirsch *et al*, 2007; Viale *et al*, 2008; Dowsett *et al*, 2009; Jones *et al*, 2010).

Nuclear Ki-67 is usually estimated as the percentage of tumour cells positively stained by immunohistochemistry. Compared with other markers of proliferation, Ki-67 proliferative activity is accurate, easy and economical to be determined, and consistent, which makes it an ideal diagnostic tool (Urruticoechea *et al*, 2005). Recently introduced image analysis techniques offer the potential for automated assessment and possibly increased precision, but this may prove difficult in heterogeneous tissues like breast carcinomas (Urruticoechea *et al*, 2005).

The aim of the present study was to assess whether automated scoring of Ki-67 proliferative activity was as accurate as visual scoring in terms of both precision and prognostic ability in primary operable invasive ductal breast cancer.

## Patients and methods

Patients presenting with invasive breast cancer at Royal Infirmary, Western Infirmary or Stobhill Hospital, Glasgow, between 1995 and 1998 were studied (*n*=379). Available clinico-pathological data included age, histological tumour type, grade, tumour size, lymph node status, oestrogen (ER) and progesterone (PR) status, type of surgery, and use of adjuvant treatment (chemotherapy, hormonal therapy and/or radiotherapy). Tumour proliferative activity was determined as Ki-67 labelling index (LI) in these patients.

Institutional Review Board approval for the use of human tissue in this study was given by the Research Ethics Committee of the North Glasgow University Hospitals NHS Trust.

### Methods

#### Tissue micro array (TMA) construction

TMAs were used in the present study. In brief, a tumour-rich area of each specimen was identified and marked by a qualified pathologist (EM), and TMAs were constructed in triplicate, using 0.6 mm^2^ cores, to account for intra-tumour disease heterogeniety (Tovey *et al*, 2006).

#### Immunohistochemistry

Ki67 immunohistochemistry was performed by established protocols in the Department of Pathology, Glasgow Royal Infirmary with appropriate positive and negative controls. Dako anti-Ki-67 (monoclonal mouse anti-human, Ki-67 antigen, clone MIB1, code M7240, DAKO, Glostrup, Denmark) was used at dilution 1 : 100 for 30 min for immunohistochemistry on a Bond Max automated slide stainer (Leica Microsystems, Wetzlar, Germany) according to the manufacturer's instructions, with Leica Envision detection system. Slides were lightly counterstained with haematoxylin, dehydrated and mounted with DPX.

#### Slide scanning and scoring

Stained slides were scanned using a Hamamatsu NanoZoomer (Hertfordshire, UK). Visualisation and automated cell counts were carried out using the Slidepath Tissue IA system version 3.0 (SlidePath's Tissue IA system, Dublin, Ireland) and visual counting of the percentage of positive cells was performed on a computer monitor.

#### Assessment of tumour proliferative activity (Ki-67)

The number of Ki-67-positive cells was counted both visually and automatically (Canna *et al*, 2008), and the percentage of positive invasive carcinoma cells, as a percentage of total tumour cells, was calculated in all three cores. As each tumour had triplicate cores, the mean count for each carcinoma was taken as a final score. A total of 65 cores were counted independently by two observers (BE and ZM) blinded to patient outcome and the other observer's score, giving an interclass correlation coefficient (ICCC) of 0.94, indicating excellent agreement. ZM subsequently scored all slides. The accuracy of scoring depends on individual cores containing a satisfactory sample of tumour cells, which was checked by a qualified pathologist (JJG).

#### Image Analysis of Ki-67 staining

For the automated determination of Ki67 LI, digitised slides were accessed through the Slidepath Image Analysis system and evaluated using the program's nuclear scoring algorithm, which quantifies nuclear staining within individual cores and derives a counting score for each target area.

Nuclei stained with polymerised diaminobenzidine and/or haematoxylin are identified and separated by a thresholding and segmentation algorithm. Using the Slidepath software, specific cell populations within a heterogeneous sample can be selected for analysis according cell nuclear area. The investigator is able to adjust the upper and lower limits of a range of acceptable nuclear areas, such that cells within the specified range are accepted for analysis, whereas those out of the range are rejected. In this way, the relatively large tumour cells can be selected over, for example, relatively much smaller inflammatory cells. However, this system does not work perfectly and inevitably there is some error in the selection process—for example, some visual artefacts may be accepted as tumour nuclei, multiple small nuclei may be confused for a single large nucleus when located close together, or obvious (to the human observer) tumour nuclei may be mistakenly rejected. These errors may represent limitations to the utility of automated image analysis.

**Figure 1:**
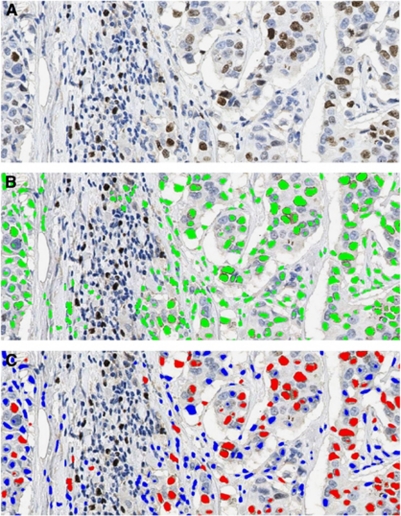
The panel (**A**) shows positive case ( × 20) by counting method, panel (**B**) shows the selected nuclei (green) and panel (**C**) shows the classification of Ki-67 within each core as positive (red) or negative (blue) based on adjustable input parameters. The colour reproduction of this figure is available at the *British Journal of Cancer* online.

Nuclear Ki-67 staining is classified as positive or negative based on observer-specified intensity thresholds. Pseudo-colours (red/blue) display these staining intensity measurements for individual nuclei, allowing thresholds to be chosen appropriately (Figure 1A–C).

Intensity thresholds were chosen for a sample of TMA cores from the whole cohort and once set they were used for analysis over the entire patient cohort without adjustment.

#### Statistical analysis

Several methods were used to examine the correlation between visual and automated Ki-67 LI in order to make comparisons with published studies easier: ICCC, Spearman's ρ and Pearson's r. Consistency between the observers was analysed using the statistic κ with values 0.40–0.59 considered to represent moderate agreement; 0.60–0.79 good, and >0.80 very good agreement (Landis and Koch, 1977). Univariate analysis with calculation of hazard ratios was performed using a Cox proportional hazards model. Deaths up to March 2010 were included in the analysis. Inter-relationships between the methods were assessed using contingency tables with the χ^2^ test for trend as appropriate. Analysis was performed using SPSS software version 18 (SPSS Inc., Chicago, IL, USA).

## Results

Clinical and pathological characteristics of patients (*n*=379) are shown in Table 1. Most were older than 50 years (69%), had a grade I or II carcinoma (54%) smaller than 2 cm (58%) with no axillary lymph node involvement (55%). A total of 225 patients (59%) had ER-positive tumours and 168 patients (44%) had PR-positive tumours. Three hundred patients (79%) had HER-2 negative tumours. In all, 184 (49%) patients received only endocrine therapy, 77 (20%) received only chemotherapy only and 95 (25%) received both.

**Figure 2:**
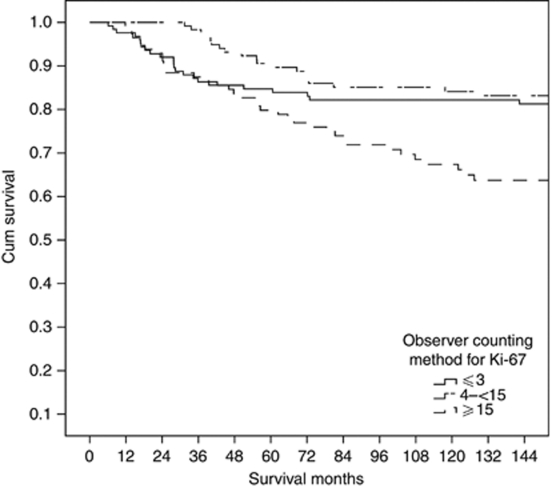
The relationship between Ki-67 assessed using tertiles by visual counting method and cancer outcome in patients with invasive ducal breast cancer.

As there are no generally accepted prognostic thresholds for Ki-67 LI, survival analysis was undertaken by tertiles. Survival curves for visually assessed Ki67 LI (Figure 2) indicated that first and second tertiles were prognostically favourable and could be considered ‘low’ with the prognostically adverse third tertile taken as ‘high’. This yielded a cutoff at 15% (<15% low, >15% high). A total of 272 (72%) patients had a carcinoma with a low Ki-67 proliferative activity using this criterion.

**Figure 3:**
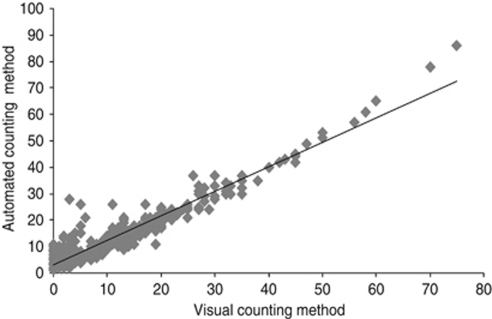
Plots and regression analysis of visual and automated counting methods for Ki-67 (rs, Spearman's Correlation).

Table 2 compares visual and automated determinations of Ki-67 LI. Of the 272 cases with a low visual Ki-67 LI, 39 (14%) cases scored high by the automated method. Of the 107 cases with a high visual Ki-67 LI, 11 (10%) scored low by the automated method. As expected, visual and automated determinations of Ki-67 LI were strongly correlated (ρ=0.87, *r*=0.94, Figure 3) with good agreement of visual and automated Ki-67 status using the cutoff described (ICCC=0.96, *P*<0.001). The κ value 0.70 also reflected good agreement.

**Figure 4:**
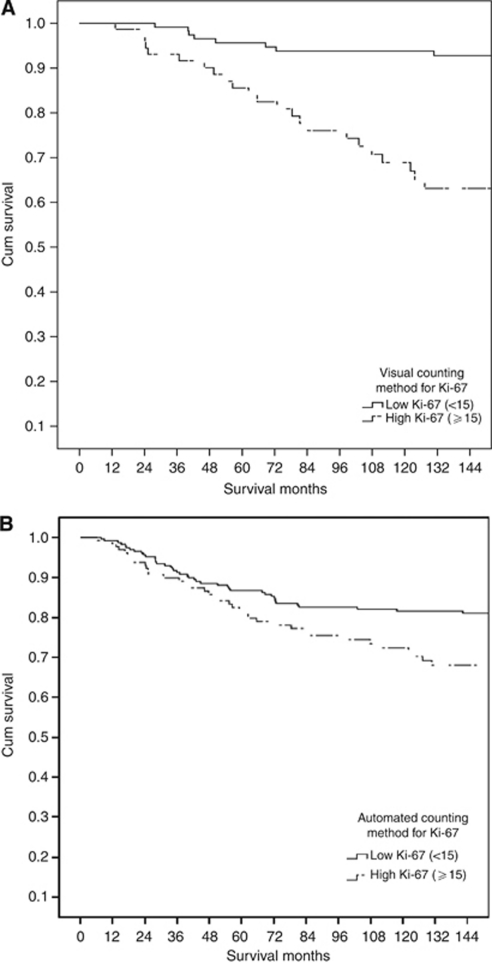
The relationship between Ki-67 assessed by visual (**A**) automated counting methods (**B**) and cancer-specific survival in patients with invasive ductal breast cancer.

The minimum follow-up was 142 months; median follow-up of survivors was 165 months. During follow up 92 patients had recurred, 15 local, 57 distant and 4 both; 163 patients died, 81 died of their cancer. Univariate relationships between survival and Ki-67 proliferative activity determined by visual and automated methods are shown in Table 3. Visual (*P*<0.01) but not automated (*P*=0.557) measurements of Ki-67 status were predictive of recurrence-free survival while both visual (<0.001) and automated (*P*<0.05) measurements were predictive of cancer-specific survival (Figure 4A and B), visual scoring achieved a higher level of statistical significance (*P*<0.001) than the automated score (*P*<0.05).

A complementary statistical approach to directly compare the prognostic value of the visual and automated methods of Ki-67 LI is to compare the areas under the receiver operator curves (AUC). Using recurrence-free and cancer-specific survival at 10 years as endpoints, the AUCs for the visual method were 0.593 (95%CI 0.524–0.662, *P*=0.008) and 0.598 (95% CI 0.523–0.672, *P*=0.009), respectively; the AUCs for the automated method were 0.536 (95% CI 0.468–0.605, *P*=0.294) and 0.555 (95% CI 0.481–0.629, *P*=0.142), respectively.

**Figure 5:**
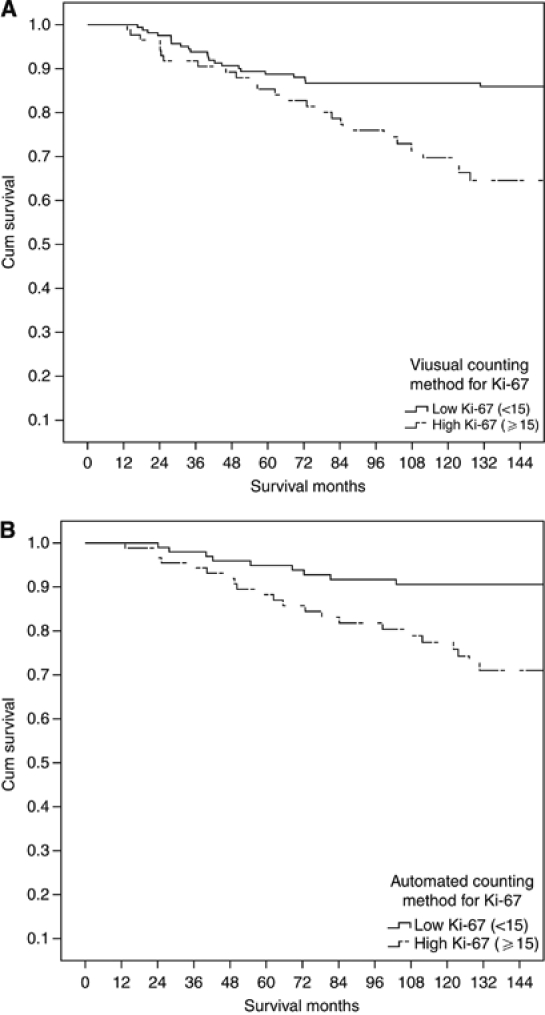
The relationship between Ki-67 assessed by visual (**A**) and automated counting methods (**B**) and cancer-specific survival in patients with invasive ductal breast cancer who received endocrine (Tamxoifen) therapy.

Cancer recurrence and cancer-specific survival on endocrine therapy (Tamoxifen) were of interest as possible indicators of the ability of different scoring methods to predict the response to Tamoxifen. Univariate survival analysis was therefore undertaken for patients with ER-positive tumour who received treatment with Tamoxifen. Recurrence-free and cancer-specific survival by Ki-67 status determined visually and automatically are shown for these patients in Table 4. Visually determined Ki-67 status predicted recurrence-free (*P*<0.01) and cancer-specific survival (*P*<0.001) in patients who received Tamoxifen, whereas the automated method (*P*<0.01) was only significantly associated with cancer-specific survival in patients who received Tamoxifen (Figure 5A and B).

## Discussion

The results of the present study show that visually assessed Ki-67 proliferative activity was associated with cancer-specific survival in patients with operable ductal breast cancer overall and in patients treated with Tamoxifen. Although Ki-67 proliferative activity assessed by the automated system was in reasonably good agreement with visual assessment, its prognostic value with respect to recurrence-free and cancer-specific survival was not as high as visual assessment. These results confirm the clinical value of visually assessed Ki-67 status and suggest that more work is required before automated assessment can be unreservedly recommended for routine clinical laboratory measurement of Ki-67 LI in patients with invasive ductal breast cancer.

Visual methods are used widely in the clinical assessment of Ki-67 proliferative activity. Centralisation of laboratory services and increasing workloads may increase interest in image analysis for the assessment of Ki-67 LI. Image analysis may possibly provide more detailed information and improve quality control (Faratian *et al*, 2009). Observer interpretations can vary, but a human observer may be better at recognising non-tumour or stromal areas in the sample than an image analysis algorithm (Konsti *et al*, 2011), which may explain why Ki-67 LI determined automatically was less predictive than visually determined Ki-67 status in the present study. Alternatively, disparities in prognostic effectiveness between visual and automated scoring in this instance may be due to the software errors in tumour cell selection described earlier.

From the literature it is clear that many cutoffs for Ki-67 LI have been used to predict cancer outcome. However, without standardisation of the methodology, such cutoffs have limited clinical value outwith specific centres. Indeed, this problem has been recognised by the International Ki-67 in Breast Cancer Working Group as they were unable to come to consensus regarding the ideal cut point(s) that might be used in routine clinical practice (Dowsett *et al*, 2011). Nevertheless, it is of interest that Ki-67 LI of between 10 and 20% have been mostly reported to be associated with cancer outcome (Stuart-Harris *et al*, 2008). In the present study, survival analysis was initially undertaken using tertiles (making no assumption of the correct prognostic cutoff point). Survival curves for these Ki-67 LI tertiles were examined (Figure 2) and indicated that first and second tertiles had similar good outcome and therefore could be considered as ‘low risk’, and the third tertile had poor outcome and therefore could be considered as ‘high risk’. This analysis yielded a cutoff at 15% staining positivity, reassuringly in the middle of the 10–20% range.

In the present study, the main question was whether the determination of Ki-67 status by visual and automated methodologies could be regarded as equivalent. The main outcome measure was the parity (or lack thereof) of automated staining assessment, compared with visual assessment methods. Agreement between the methods was good, which was determined using standard statistical approaches. However, the two methods yielded significantly different prognoses with respect to recurrence-free and cancer-specific survival using Cox regression analysis and Receiver operator characteristics.

In the present study, 14% of patients with low visual Ki-67 status were in the high Ki-67 group by automated assessment. Dowsett and co-workers pointed out that negative nuclei determine the overall population for calculating the proportion of Ki-67-positive cells, and that weak counterstaining can therefore result in an overestimation of the Ki-67 index. Thus, it is important to optimise the degree of counterstaining (Dowsett *et al*, 2011). It is possible that the counterstain used in this study was slightly weak, therefore resulting in an underestimation of negative nuclei and, hence, an overestimation of the Ki-67 index in some cases. Such a scenario might explain some of the observed discrepancies. Moreover, a few discrepant cases were associated with section damage, dye precipitates, imperfect (out-of-focus) scanning or cytoplasmic staining. So, although automated Ki-67 LI has some promise to replace the visual method in the routine clinical pathology laboratory, considerable care will be required to generate reliable clinical measurements.

Other studies have investigated the automated assessment of Ki-67 status in breast cancer (Fasanella *et al*, 2011; Konsti *et al*, 2011). Correlations between visual and automated assessment were similar (*r*=0.94 for this study, 0.85 Fasanella; κ 0.70 for this study, 0.57 Konsti), despite the use of different image analysis systems. Fasanella *et al* (2011) did not examine the relationships between Ki-67 proliferative activity and survival. In a more comparable study, Konsti *et al* (2011) reported that automated assessment of Ki-67 proliferative activity had prognostic value in 1334 breast cancer patients, but did not examine the survival relationships in the subgroup of patients who received Tamoxifen.

In conclusion, the present study does show good agreement between visual and automated assessment of Ki-67 proliferative activity in invasive breast cancer, and that automated assessment of Ki-67 LI would appear to be comparable to visual Ki-67 LI. However, automated Ki-67 LI assessment was inferior in predicting cancer survival in patients with breast cancer, including patients who received Tamoxifen. Visually determined Ki-67 status was better, and therefore, although automated assessment of Ki-67 proliferative activity may have a role in clinical assessment of breast cancer, careful validation remains necessary.

## Figures and Tables

**Table 1:** The clinico-pathological characteristics of patients with primary operable invasive ductal breast cancer (*n*=379)

**Clinico-pathological characteristics**	**Patients (*n*)**
Age (⩽50/>50 years)	117 (31%)/262 (69%)
Size (⩽20/21–50/>50 mm)	219 (58%)/149 (39%)/11 (3%)
Grade (I/II/III)	59 (16%)/143 (38%)/177 (47%)
Involved lymph node (0/1–3/>3)	207 (55%)/106 (28%)/63 (17%)
Oestrogen receptor status (ER−/ER+)	148 (39%)/225 (59%)
Progesterone receptor status (PR−/PR+)	208 (55%)/168 (44%)
HER-2 status (HER-2−/HER-2+)	300 (79%)/71 (19%)
Ki-67 (low/high)	272 (72%)/107 (28%)
Loco-regional treatment (Lumpectomy+radiotherapy/mastectomy+radiotherapy)	153 (40%)/226 (60%)
Systemic treatment (ER-based treatment) (hormonal/hormonal+chemotherapy/chemotherapy/none)	184 (49%)/77 (20%)/95 (25%)/18 (5%)

**Table 2:** The comparison between visual counting assessment and automated counting assessment for Ki-67 for patients with invasive ductal breast cancer

	**Automated counting assessment**			
		**Low**	**High**	
**Visual counting assessment**	Low	233 (86%)	39 (14%)	272
	High	11 (10%)	96 (90%)	107
		244	135	379

**Table 3:** The relationship between visual counting method and automated counting method of Ki-67 status and recurrence-free and cancer-specific survival in patients with invasive ductal breast cancer (univariate analysis) (*n*=379)

	**Recurrence-free survival**		**Cancer-specific survival**	
	**Hazard ratio (95% CI)**	***P*-value**	**Hazard ratio (95% CI)**	***P*-value**
Visual counting method	1.95 (1.23–3.10)	0.005	2.43 (1.57–3.76)	<0.001
Automated counting method	1.12 (0.76–1.65)	0.557	1.75 (1.13–2.70)	0.012

Abbreviation: CI=confidence interval.

**Table 4:** The relationship between visual counting method and automated counting for of Ki-67 status, recurrence-free and cancer-specific survival in patients with invasive ductal breast cancer who received Tamoxifen (univariate analysis) (*n*=201)

	**Recurrence-free survival**		**Cancer-specific survival**	
	**Hazard ratio (95% CI)**	***P*-value**	**Hazard ratio (95% CI)**	***P*-value**
Visual counting	3.85 (1.77–8.34)	0.001	5.55 (2.57–11.98)	<0.001
Automated counting	1.65 (0.78–3.51)	0.189	3.16 (1.50–6.65)	0.003

Abbreviation: CI=confidence interval.

## References

[bib1] Al Murri AM, Hilmy M, Bell J, Wilson C, McNicol A, Lannigan A, Doughty JC, McMillan DC (2008) The relationship between the systemic inflammatory response, tumour proliferative activity, T-lymphocytic and macrophage infiltration, microvessel density and survival in patients with primary operable breast cancer. Br J Cancer 99: 1013–1019 18797461 10.1038/sj.bjc.6604667 PMC2567062

[bib2] Cancerstats (2008) http://info.cancerresearchuk.org/cancerstats/types/breast/incidence. Accessed 25 June 2010

[bib3] Canna K, Hilmy M, McMillan DC, Smith GW, McKee RF, McArdle CS, McNicol A (2008) The relationship between tumour proliferative activity, the systemic inflammatory response and survival in patients undergoing curative resection for colorectal cancer. Colorectal Dis 10: 663–667 18005189 10.1111/j.1463-1318.2007.01416.x

[bib4] de Azambuja E, Cardoso F, de Castro GJ, Colozza M, Mano MS, Durbecq V, Sotiriou C, Larsimont D, Piccart-Gebhart MJ, Paesmans M (2007) Ki-67 as prognostic marker in early breast cancer: a meta-analysis of published studies involving 12,155 patients. Br J Cancer 96: 1504–1513 17453008 10.1038/sj.bjc.6603756 PMC2359936

[bib5] Dowsett M, A’hern R, Salter J, Zabaglo L, Smith IE (2009) Who would have thought a single Ki67 measurement would predict long-term outcome? Breast Cancer Res 11(Suppl 3): S15 20030866 10.1186/bcr2434 PMC2797695

[bib6] Dowsett M, Nielsen TO, A’hern R, Bartlett J, Coombes RC, Cuzick J, Ellis M, Henry NL, Hugh JC, Lively T, McShane L, Paik S, Penault-Llorca F, Prudkin L, Regan M, Salter J, Sotiriou C, Smith IE, Viale G, Zujewski JA, Hayes DF (2011) Assessment of Ki67 in Breast Cancer: Recommendations from the International Ki67 in Breast Cancer Working Group. J Natl Cancer Inst 103(22): 1656–1664 21960707 10.1093/jnci/djr393 PMC3216967

[bib7] Faratian D, Kay C, Robson T, Campbell FM, Grant M, Rea D, Bartlett JMS (2009) Automated image analysis for high-throughput quantitative detection of er and pr expression levels in large-scale clinical studies: the team trial experience. Histopathology 55: 587–593 19912364 10.1111/j.1365-2559.2009.03419.x

[bib8] Fasanella S, Leonardi E, Cantaloni C, Eccher C, Bazzanella I, Aldovini D, Bragantini E, Morelli L, Cuorvo LV, Ferro A, Gasperetti F, Berlanda G, Dalla Palma P, Barbareschi M (2011) Proliferative activity in human breast cancer: ki-67 automated evaluation and the influence of different ki-67 equivalent antibodies. Diagn Pathol 6(Suppl 1): S7 21489202 10.1186/1746-1596-6-S1-S7 PMC3073225

[bib9] Fitzgibbons PL, Page DL, Weaver D, Thor AD, Allred DC, Clark GM, Ruby SG, O’Malley F, Simpson JF, Connolly JL, Hayes DF, Edge SB, Lichter A, Schnitt SJ (2000) Prognostic factors in breast cancer. College of American Pathologists Consensus Statement 1999. Arch Pathol Lab Med 124: 966–978 10888772 10.5858/2000-124-0966-PFIBC

[bib10] Goldhirsch A, Wood WC, Gelber RD, Coates AS, Thürlimann B, Senn H (2007) Progress and promise: highlights of the international expert consensus on the primary therapy of early breast cancer 2007. Ann Oncol 18: 1133–1144 17675394 10.1093/annonc/mdm271

[bib11] Jones RL, Salter J, A’Hern R, Nerurkar A, Parton M, Reis-Filho JS, Smith IE, Dowsett M (2010) Relationship between oestrogen receptor status and proliferation in predicting response and long-term outcome to neoadjuvant chemotherapy for breast cancer. Breast Cancer Res Treat 119: 315–323 19247830 10.1007/s10549-009-0329-x

[bib12] Jung S, Han W, Lee JW, Ko E, Kim E, Yu J, Moon H, Park IA, Oh D, Im S, Kim T, Hwang K, Kim S, Noh D (2009) Ki-67 expression gives additional prognostic information on st. gallen 2007 and adjuvant! Online risk categories in early breast cancer. Ann Surg Oncol 16: 1112–1121 19219507 10.1245/s10434-009-0334-7

[bib13] Konsti J, Lundin M, Joensuu H, Lehtimäki T, Sihto H, Holli K, Turpeenniemi-Hujanen T, Kataja V, Sailas L, Isola J, Lundin J (2011) Development and evaluation of a virtual microscopy application for automated assessment of ki-67 expression in breast cancer. BMC Clin Pathol 11: 3 21262004 10.1186/1472-6890-11-3 PMC3040126

[bib14] Landis JR, Koch GG (1977) The measurement of observer agreement for categorical data. Biometrics 33: 159–174 843571

[bib15] McCormick D, Chong H, Hobbs C, Datta C, Hall PA (1993) Detection of the ki-67 antigen in fixed and wax-embedded sections with the monoclonal antibody mib1. Histopathology 22: 355–360 8514278 10.1111/j.1365-2559.1993.tb00135.x

[bib16] Parkin DM, Bray F, Ferlay J, Pisani P (2001) Estimating the world cancer burden: globocan 2000. Int J Cancer 94: 153–156 11668491 10.1002/ijc.1440

[bib17] Scott RJ, Hall PA, Haldane JS, van Noorden S, Price Y, Lane DP, Wright NA (1991) A comparison of immunohistochemical markers of cell proliferation with experimentally determined growth fraction. J Pathol 165: 173–178 1683905 10.1002/path.1711650213

[bib18] Stuart-Harris R, Caldas C, Pinder SE, Pharoah P (2008) Proliferation markers and survival in early breast cancer: a systematic review and meta-analysis of 85 studies in 32,825 patients. Breast 17(4): 323–334 18455396 10.1016/j.breast.2008.02.002

[bib19] Tovey SM, Dunne B, Witton CJ, Cooke TG, Bartlett JMS (2006) Her4 in breast cancer: comparison of antibodies against intra- and extra-cellular domains of her4. Breast Cancer Res 8: R19 16606438 10.1186/bcr1394 PMC1557726

[bib20] Urruticoechea A, Smith IE, Dowsett M (2005) Proliferation marker Ki-67 in early breast cancer. J Clin Oncol 23(28): 7212–7220 16192605 10.1200/JCO.2005.07.501

[bib21] Viale G, Giobbie-Hurder A, Regan MM, Coates AS, Mastropasqua MG, Dell’Orto P, Maiorano E, MacGrogan G, Braye SG, Ohlschlegel C, Neven P, Orosz Z, Olszewski WP, Knox F, Thürlimann B, Price KN, Castiglione-Gertsch M, Gelber RD, Gusterson BA, Goldhirsch A (2008) Prognostic and predictive value of centrally reviewed ki-67 labeling index in postmenopausal women with endocrine-responsive breast cancer: results from breast international group trial 1–98 comparing adjuvant tamoxifen with letrozole. J Clin Oncol 26: 5569–5575 18981464 10.1200/JCO.2008.17.0829 PMC2651094

[bib22] Yerushalmi R, Woods R, Ravdin PM, Hayes MM, Gelmon KA (2010) Ki67 in breast cancer: prognostic and predictive potential. Lancet Oncol 11: 174–183 20152769 10.1016/S1470-2045(09)70262-1

